# Simulation of Flow Around a Finite Rectangular Prism: Influence of Mesh, Model, and Subgrid Length Scale

**DOI:** 10.3390/e27010065

**Published:** 2025-01-13

**Authors:** Xutong Zhang, Maxime Savoie, Mark K. Quinn, Ben Parslew, Alistair Revell

**Affiliations:** Department of Mechanical and Aerospace Engineering, The University of Manchester, Manchester M1 3PL, UK; savoie.maxime@outlook.com (M.S.); mark.quinn@manchester.ac.uk (M.K.Q.); ben.parslew@manchester.ac.uk (B.P.); alistair.revell@manchester.ac.uk (A.R.)

**Keywords:** RANS, hybrid RANS LES, DDES, mesh refinement, grey area, subgrid length scale, external aerodynamics

## Abstract

This study investigates the flow field around a finite rectangular prism using both experimental and computational methods, with a particular focus on the influence of the turbulence approach adopted, the mesh resolution employed, and different subgrid length scales. Ten turbulence modelling and simulation approaches, including both ‘scale-modelling’ Reynolds-Averaged Navier–Stokes (RANS) models and ‘scale-resolving’ Delayed Detached Eddy Simulation (DDES), were tested across six different mesh resolutions. A case with sharp corners allows the location of the flow separation to be fixed, which facilitates a focus on the separated flow region and, in this instance, the three-dimensional interaction of three such regions. The case, therefore, readily enables an assessment of the ‘grey-area’ issue, whereby some DDES methods demonstrate delayed activation of the scale-resolving model, impacting the size of flow recirculation. Experimental measurements were shown to agree well with reference data for the same geometry, after which particle image velocimetry (PIV) data were gathered to extend the reference dataset. Numerical predictions from the RANS models were generally quite reasonable but did not show improvement with further refinement, as one would expect, whereas DDES clearly demonstrated continuous improvement in predictive accuracy with progressive mesh refinement. The shear-layer-adapted (SLA) subgrid length scale ($\Delta_{\mathrm{SLA}}$) displayed consistently superior performance compared to the more widely used length scale based on local cell volume, particularly for moderate mesh resolutions commonly employed in industrial settings with limited resources. In general, front-body separation and reattachment exhibited greater sensitivity to mesh refinement than wake resolution. Finally, in order to correlate the observed DDES mesh requirements with the observations from the converged RANS solutions, an approximation for the Taylor microscale was explored as a potential tool for mesh sizing.

## 1. Introduction

Commercial freight vehicles remain one of the major sources of ${\mathrm{CO}}_{2}$ emissions globally, contributing around a quarter of road transport emissions and 6% of total ${\mathrm{CO}}_{2}$ emissions across Europe [1]. At 70 mph, 65% of a vehicle’s total energy is used to overcome aerodynamic drag [2]. Increased environmental awareness places aerodynamic drag under even greater scrutiny than before and has motivated a renewed interest in vehicle aerodynamics in recent years. Computational fluid dynamics (CFD) is routinely used by industry to evaluate external aerodynamic designs due to its flexibility and cost-effectiveness compared to experimental testing. However, the accurate prediction of turbulent flow around complex geometries at high Reynolds numbers remains a challenge, despite the more regular use of scale-resolving methods capable of greater accuracy [3,4,5,6].

A key problem in automotive aerodynamics is the trade-off between simulation accuracy and geometry complexity. Previous studies [7,8,9] have demonstrated that, despite currently remaining the most widely used approaches in industry, standard Reynolds-Averaged Navier–Stokes (RANS) models routinely overestimate aerodynamic drag, particularly the linear Eddy Viscosity Models (EVMs). Classical scale-resolving methods, such as Large Eddy Simulation (LES), offer greater accuracy but are often too costly for practical use. Therefore, Hybrid RANS–LES (HRL) methods, including Detached Eddy Simulation (DES), Partially Averaged Navier–Stokes (PANS) [10], Scale-Adaptive Simulation (SAS) [11], and Very Large Eddy Simulation (VLES) [12], are often selected as more practical options. In particular, these approaches are particularly attractive since they can be applied throughout a computational domain and left to switch automatically between RANS and LES modes, according to functions that estimate turbulence levels over different regions of the domain. They are, therefore, often known as ‘global’ or ‘seamless’ approaches.

Owing to their ease of use, seamless HRL methods are widely used in industry, with DES being particularly popular. Initially developed to address the limitations of traditional RANS models, DES was later refined into Delayed Detached Eddy Simulation (DDES) to improve boundary layer shielding [13] and was modified further for use with the Shear Stress Transport (SST) model by [14]. Additionally, the Improved DDES (IDDES) model was introduced, combining DDES with Wall-Modelled LES (WMLES) for greater flexibility in handling complex geometries and flow conditions [15].

Since the inception of the DES method, the main challenge and subsequent focus have been on the ability of the model to switch from representing turbulence entirely via a RANS approach to one where turbulence has both a modelled and a resolved component, as is the case with HRL methods (see, for instance, the work and adjustments proposed in [16,17,18,19].) Indeed, there is often a region of the flow where the switch to the LES mode is not complete, which leads to erroneous accounting of turbulence. This so-called ‘grey-area’ problem is related to excessive eddy viscosity generated by the RANS part of the model, which takes time to fully dissipate once the LES mode has been activated. The excessive eddy viscosity acts to reduce local velocity fluctuations and reduces the levels of resolved Turbulence Kinetic Energy (TKE). Two approaches are commonly employed to address this issue: (1) the addition of artificial turbulence in the region of interest, and (2) the local reduction in eddy viscosity based on knowledge of some kind that the flow should be predominantly resolved at that point. The former choice requires, among other options, the addition of synthetic turbulence [20,21]. The latter can be achieved by the careful adjustment of the subgrid length scales (SLSs), as in [16,18,19,22,23], and is therefore favourable in this context, as it preserves the ‘seamless’ nature of the methodology.

This work explores the sensitivity of three aspects of DDES: the underlying turbulence model, the subgrid length scale, and the mesh resolution. The most common options for the SLS are based on either the cube root of the volume of the local mesh cell ($\Delta_{\mathrm{vol}}$) or its largest coordinate dimension ($\Delta_{\max}$). In 2007, a vorticity direction-based SLS, $\Delta_{\omega }$, was proposed by Chauvet et al. [16], which was intended to reduce the length scale to a two-dimensional version, i.e., $\sqrt{\Delta_{x}\Delta_{y}}$, in regions where the scale resolution is primarily two-dimensional, the ‘quasi-2D region’, where *x* and *y* denote the streamwise and wall-normal directions. In 2015, based on the idea of $\Delta_{\omega }$, Mockett et al. developed ${\tilde{\Delta }}_{\omega }$ by replacing $\Delta_{\mathrm{vol}}$ in the equation with $\Delta_{\max}$ [22]. Shur et al. [18] later extended this by proposing a formulation that forces the turbulence model to ‘switch off’ the RANS model in the quasi-2D region. This approach became known as the shear-layer-adapted (SLA) model, denoted as $\Delta_{\mathrm{SLA}}$. Since its introduction, the SLA method has been applied across various domains, ranging from separated flows over simple 2D geometries in both subsonic and supersonic regimes using DDES [24,25,26] and IDDES [27] to jet noise predictions [28]. Applications also extend from low-Mach wind turbine flows [29] to high-Mach reentry vehicles in aerospace engineering [30]. However, its application to automotive aerodynamics remains relatively limited compared to conventional DDES, and there is a lack of a systematic analysis of mesh resolution and its impact on SLA performance.

In general, there has been significant growth in recent years in the application of HRL methods to the study of ground vehicle aerodynamics, although relatively little of this has specifically focused on freight transport vehicles when compared to the focus on more conventional vehicle geometries, such as the Ahmed body [31,32,33,34] and DrivAer [35,36]. The Ground Transport System (GTS) is a counterexample that has attracted some attention, representing a simplified truck model and used as a basis for exploring drag-reduction strategies [37]. The GTS geometry, which has been subject to both experimental [38] and computational work [39,40], has a taller, longer body and incorporates a curved front corner, representing the typical aerodynamic fairing on cab-trailer configurations. This feature, while relevant, introduces additional complexity on account of the additional challenge associated with the correct prediction of boundary layer separation from a smooth surface, as demonstrated by Rao et al. [39], wherein the LES results indicated a flow separation not clearly observed in the experimental data.

The present work sought instead to focus on the flow around a cuboid (a wagon), originally defined and tested by Li et al. [41], to investigate the drag reduction of railway freight. As a 5.85:3.15:1 cuboid, this model shares similarities with heavy commercial vehicles but with sharp corners. Therefore, the flow separation is fixed at both the leading and trailing edges, which focuses the challenge on predicting the form and magnitude of recirculation regions over the top, sides, and rear, as well as the three-dimensional interactions between them. Moreover, since it has been accessed by different experimental techniques, including stereoscopic particle image velocimetry (PIV) [41,42], it provides an interesting benchmark for numerical results.

In this paper, we report on a combined experimental and numerical study of the turbulent flow around a 5.85:3.15:1 wagon. The focus is on the assessment of several turbulence models, both RANS and DDES methods, to investigate their sensitivities to the underlying mesh, with the aim of developing a clearer understanding of precisely where refinement is needed and for what gain.

## 2. Methodology

### 2.1. Case Description

The geometry is defined as a 0.5m(L)×0.219m(H)×0.0855m(W) cuboid. The experiments were performed in an open-circuit wind tunnel at the University of Manchester with a 5m(L)×0.9m(H)×0.9m(W) test section. The flow speed was $U_{\infty }=18\;m/s$, with a corresponding Reynolds number of $Re_{W}=10^{5}$ based on the model width. The geometry was mounted 0.233m downstream from the leading edge (LE) of the splitter plate with a 0.014m ride height. Force balance, pitot tube, and PIV methods were used to acquire both the integral and flow field quantities. For reference, a full-size freight transport vehicle (width 2.5 m) may experience Reynolds numbers in excess of $50\times 10^{6}$, but this was beyond the capability of the current experimental facility. Despite this difference, the geometry in question is unlikely to exhibit significant Reynolds number dependence due to the flow separation being fixed at the point of the sharp corners. Furthermore, modelling issues related to the ‘grey-area’ problem are known to reduce with increasing Reynolds numbers, and as such, a focus on lower Re provides the basis for a more stringent assessment of these models.

### 2.2. Experimental Setup

The experiments were performed in the University of Manchester’s open-circuit “BOB” wind tunnel. The speed was adjusted with a variable-pitch axial fan, driven by a three-phase electric motor, pulling air through the test section. Upstream of the test section were two turbulence-reducing screens, followed by a long-cell honeycomb structure to straighten the flow, leading to a 5:1 contraction section and finally to the test section.

A hot-wire anemometer was used in the free stream to measure the turbulence intensity of the flow in the test section. Tests were performed at the wind tunnel’s minimum and maximum speeds. The tests took place on two different days and consisted of at least three runs in which the velocity was averaged at least three times for 30 s. In both cases, the turbulence intensity was measured to be less than 1%.

**Figure 1:**
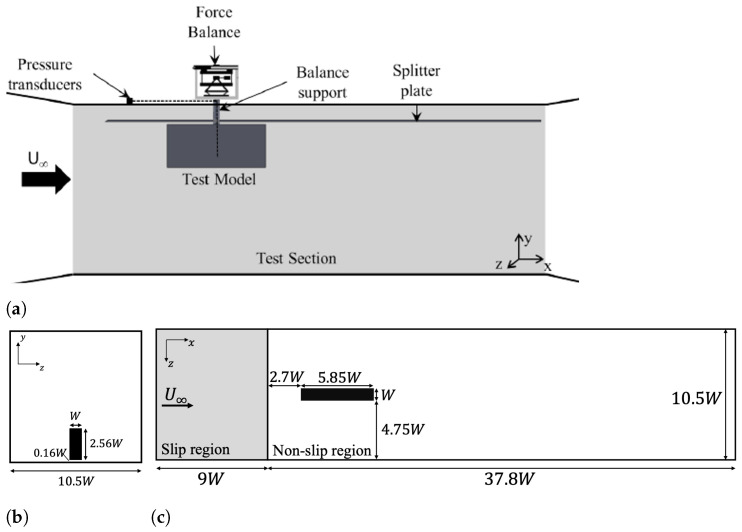
(**a**) Experimental setup and CFD domain, (**b**) top view, and (**c**) front view.

The experimental setup and CFD domain are shown in Figure 1. The test model is located 2.1 W downstream of the splitter leading edge in the wind tunnel. The blockage ratio of 2.7%, calculated by the ratio of the cross-sectional areas of the model over the working test section, $B=A_{\mathrm{model}}/A_{\mathrm{WT}}$, is less than 5%, which is generally accepted to be below the threshold for which a wind tunnel correction is required [43].

In this work, pressure measurements taken via pressure transducers were combined with signals coming from the force balance. A PC running the LabVIEW 2016 DAQ programme [44] was used to control the wind tunnel’s speed and acquire the data. The wind tunnel’s dynamic pressure was calculated using the velocity derived from the measurement of a pitot tube at the centre of the test section upstream of the splitter plate. The wind tunnel’s speed was controlled by pitching the blades of a fan that pulled the air through the test section.

Pressure measurements were carried out using 24 NXP MPXV7002DP dual-port differential pressure transducers (NXP Semiconductors, Eindhoven, The Netherlands) [45]. The sensors were connected to the PC where a LabVIEW programme allowed us to zero them before each run and read and save the data. The sensitivity of the sensor was 1 V/kPa with a 5 V power supply.

Stereoscopic particle image velocimetry (S-PIV) was also performed. The arrangement included two identical cameras and a dual-cavity Nd:YAG laser mounted on remote-controlled traverses. Two Imager SX 4M (2360 × 1776) CCD cameras (LaVision GmbH, Gottingen, Germany) cameras with 25 mm fixed focal length lenses were used, allowing a field of view of approximately 0.4 m × 0.4 m. The laser system was a dual-cavity Litron nano L-PIV (Litron Lasers Ltd., Rugby, UK) with a maximum output energy of 200 mJ operating at a wavelength of 532 nm. The flow was seeded using Di-Ethyl-Hexyl-Sebacate (DEHS), which produces particle sizes between 0.2 µm and 0.3 µm. The DEHS was aerosolised using a TSI 9306 atomizer (TSI Inc., Shoreview, MN, USA) and passed into an array of perforated tubes located vertically in front of the wind tunnel inlet to seed the entire test section. The measuring plane was illuminated by a laser sheet with a waist of approximately 2 mm. The time interval between PIV images ($\delta_{t}$) was set at 90 µs so that the displacement was large enough for accurate velocity field estimations. The time-averaged velocity fields were processed using LaVision DaVis 10.1 software [46], and the velocity vectors were estimated on a 32 × 32-pixel interrogation window with a 75% overlap.

**Figure 2:**
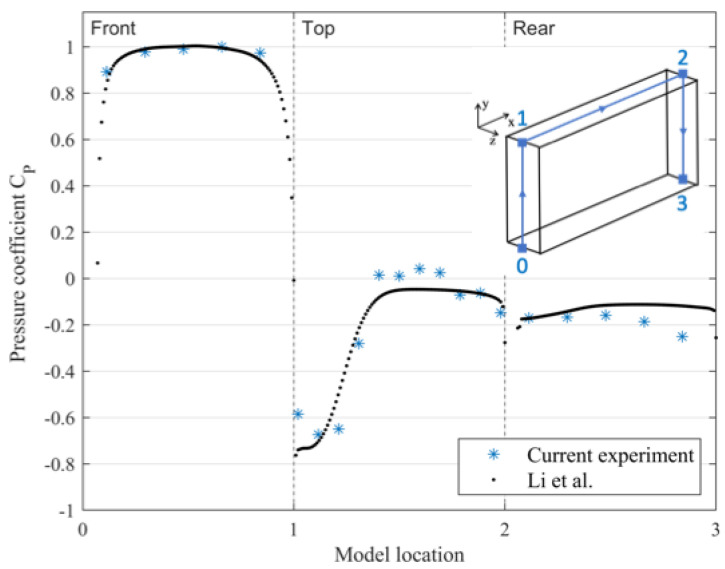
Time-averaged surface pressure coefficient ($C_{P}$) on the mid-section line of the front, top, and base surfaces. Results are compared with experimental data from Li et al. [42].

To validate the experimental setup, the time-averaged surface pressure coefficients of the mid-section line for different surfaces were compared to a previous experiment for the same geometry and Reynolds number [42], as shown in Figure 2. The time-averaged surface pressure coefficients showed good agreement with this dataset. Some slight differences can be seen at the top and rear surfaces, but this is largely within the expected range of the experimental error. Despite these differences, the locations of the flow separation and their corresponding reattachment are similar for both experiments, particularly considering that various differences exist between the two experiments.

### 2.3. CFD Setup

The CFD simulations were set up based on the experimental configuration shown in Figure 1. The inlet was defined as a uniform velocity inlet with $U_{\infty }=18\;m/s$, while the outlet was specified as a pressure outlet. The wagon surface was modelled as a no-slip wall. To minimise the influence of potential reverse flow at the inlet boundary, a slip region extending 9 W downstream from the inlet patch was applied, as shown in Figure 1c. Following this, a no-slip floor region was introduced to replicate the splitter plate used in the experiments. The wagon was positioned 2.1 W downstream from the start of the no-slip floor region with a ride height of 0.16 W. The side and top walls of the computational domain were treated as symmetry boundary conditions to reduce the cell count by eliminating the need for prism layers along these surfaces. Based on the turbulent boundary layer thickness calculated along a flat plate [47]:

(1) δ99(x)≈0.38xRex1/5,

where the boundary layer thickness over the side and top walls is estimated to be no less than 5% of the wind tunnel’s width and height. Consequently, the simplification of treating these boundaries as symmetry planes was deemed reasonable.

For the test matrix, as presented in Table 1, nine turbulence models, including the widely used RANS and HRL methods, were assessed on six different meshes using the open-source software OpenFOAM v6. Three different RANS methods were tested: the Spalart–Allmaras (SA) method [48], the k-ω SST (SST) method [49], and the Elliptic-Blending Reynolds Stress Model (EBRSM) developed by Manceau [50]. Meanwhile, the k-ω SST DDES (SST-DDES) and Spalart–Allmaras DDES (SA-DDES) models were each tested with three different SLSs: the cube root volume ($\Delta_{\mathrm{vol}}$), the max cell size ($\Delta_{\max}$), and the shear layer adapted ($\Delta_{\mathrm{SLA}}$) developed by Shur et al. [18]. The details of the $\Delta_{\mathrm{SLA}}$ can be found in Appendix A. Finally, in order to provide a reference numerical result, a finer mesh was developed for a reference Large Eddy Simulation.

All cases in this study were solved using the PISO algorithm with an Euler (1st-order implicit) temporal scheme and a linear upwind (2nd-order upwind-biased) spatial scheme, which are CFD settings commonly employed in industry. The time step was set to $\Delta t=10^{-5}\;s$. The corresponding Courant–Friedrichs–Lewy (CFL) number, defined as $\mathrm{CFL}=\frac{\bar{U}}{\Delta }\Delta t$, where *U* is the local velocity and Δ is the local grid size, had a global maximum of CFLmax≈10 and an average of CFLavg<0.1 for all meshes.

To enhance simulation stability, the RANS cases were initialised using the potential flow, while the DDES cases were initialised using the RANS results from the C mesh. LES initialisation was carried out using the F mesh SST-SLA results, and a 1st-order upwind scheme was employed at the initial stage to ensure its stability. After 200 time steps (0.002s, ∼0.036 convective time units), the scheme was reverted to 2nd order to ensure the accuracy of the simulation.

The Convective Time Unit (CTU), calculated based on the free-stream velocity $U_{\infty }=18\;m/s$ and vehicle length L = 0.5 m, was CTU=0.056s. In this study, all simulations (RANS, DDES, and LES) were averaged over approximately 85CTUs, starting from t≈5 CTUs, to exclude the initial transient behaviour, meeting industry standard practice [6].

Simulations were conducted on a mix of Tier 1 and 2 facilities. Differences in hardware and software compilation make a comprehensive comparison of computational resources difficult to obtain. Qualitatively, for a given cell count per processor, LES was approximately 10% more expensive than DDES with the $\Delta_{\mathrm{vol}}$ and $\Delta_{\max}$ models, primarily due to the higher number of sub-iterations required. $\Delta_{\mathrm{SLA}}$ had a similar computational cost to LES for the same mesh due to the added complexity of its SLS model. Both unsteady RANS models required less than half the computational cost of DDES per iteration due to lower sub-iteration requirements; their overall costs were also substantially lower due to reduced time-averaging.

### 2.4. Mesh Design

**Figure 3:**
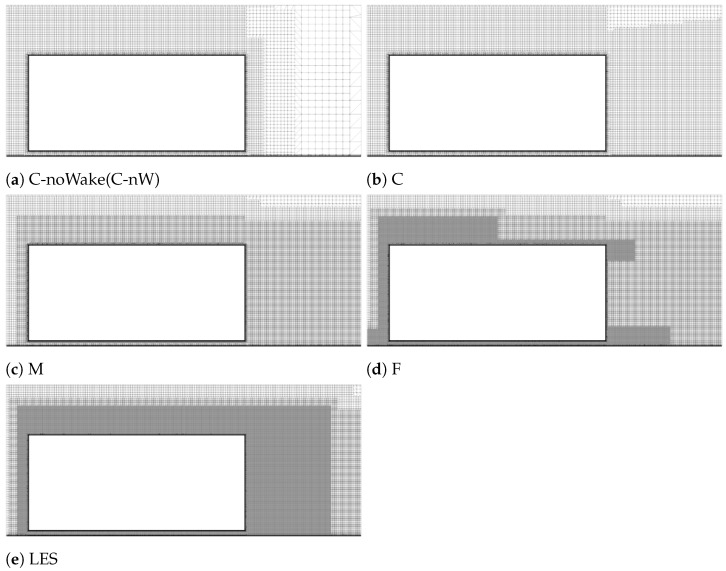
Different levels of mesh refinement designed on the symmetry (z=0) plane.

In this study, we used Star-CCM+ 2021.1 [51] to generate octree meshes. While Star-CCM+ may not be essential for the relatively simple geometry used here, we chose it to reflect common industry practices, as Star-CCM+ is widely employed in industry for its robustness in handling complex geometries. Mesh refinement plays a critical role in determining the accuracy and efficiency of aerodynamic simulations. In this study, the M mesh (Figure 3c) served as the baseline configuration, characterized by uniform refinement around the geometry and wake refinement in the downstream region. Beyond the baseline mesh configuration, a number of common incremental changes were made in a way that mimicked common practice.

The C (Figure 3c) and F (Figure 3d) meshes represent variations in refinement levels: the C mesh is coarser, achieved by removing some refinements according to standard industry practices, whereas the F mesh is finer, with additional refinement applied in regions of high-velocity gradients.

Two further coarsened meshes, C-WM and C-nW (C-noWake, Figure 3a), were introduced. The C-WM (wall-modelled) mesh was derived from the C mesh, featuring a wall-modelled prism layer with a maximum wall-normal distance of $y_{\max}^{+}\approx 100$, optimizing it for wall modelling. Meanwhile, the C-nW mesh is a version of the C mesh with wake refinement removed entirely, reducing the resolution in the wake region.

Finally, the F mesh underwent further refinement for use as a Large Eddy Simulation (LES, Figure 3e) benchmark. This refined F mesh included an increased surface mesh resolution and extensive uniform wake refinement. It was employed with the *k*-equation model and $\Delta_{\mathrm{vol}}$ SLS for LES benchmarking.

The mesh parameters and corresponding non-dimensionalised first-layer quantities are given in Table 2. All meshes, except for the C-WM, have an identical prism layer setup over both the wagon and the floor surfaces, with a $y^{+}$ value smaller than 1. The C-WM mesh has its prism layer 1st layer thickness satisfying $y^{+}$ around 30, which meets the requirement for applying wall functions. For the surface mesh, the baseline M mesh is designed by using 500 points to resolve the whole length of the wagon, which results in corresponding $x^{+}$ and $z^{+}$ values around 20.

### 2.5. Uncertainty Analysis of Experiment

The total uncertainty estimate is the combination of systematic and random errors. A systematic error refers to the portion of the total error that remains consistent across multiple runs, while a random error fluctuates unpredictably during testing [52].

In this study, the force balance systematic error was accounted for through a comprehensive calibration process, which identified sensitivities along the three axes, establishing a calibration matrix. The instrumentation’s systematic error was determined based on the individual specifications provided by the equipment suppliers. The random error was estimated by repeating the same type of run multiple times and analysing the variability in the results.

According to the equation of the drag coefficient calculation,

(2) $$C_{D}=\frac{F_{D}}{q\times A},$$

where $F_{D}$ is the drag, *q* is the dynamic pressure, and *A* is the reference area of the calculation. By using the Taylor series method for error propagation, the systematic standard uncertainty is given by

(3) $$s_{C_{D}}^{2}={\left(\frac{\partial C_{D}}{\partial F_{D}}b_{F}D\right)}^{2}+{\left(\frac{\partial C_{D}}{\partial q}b_{q}\right)}^{2}={\left(\frac{1}{qA}b_{F}D\right)}^{2}+{\left(-\frac{F_{D}}{q^{2}A}b_{q}\right)}^{2},$$

where $b_{F}D$ and $b_{q}$ are the elemental systematic error sources. Meanwhile, the random error $r_{D}$ is calculated as the square root of the standard deviation of the test results, and the final uncertainty $u_{C_{D}}$ is

(4) $$u_{C_{D}}=\sqrt{s_{C_{D}}^{2}+r_{D}^{2}}.$$

## 3. Results and Discussion

In the following, we compare different flow features, starting with forces, followed by the global flow field, velocity field, and finally, turbulent quantities.

### 3.1. Drag Coefficient

**Figure 4:**
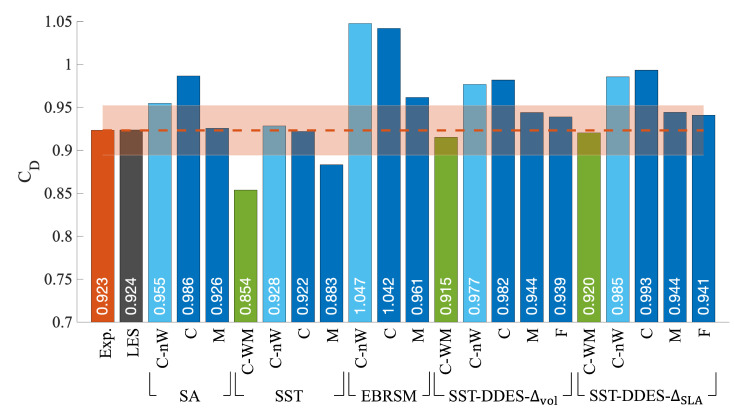
Drag coefficient comparison between experimental results (orange dashed line) with error bar (orange shaded region) and CFD results using different meshes and turbulence models.

The predicted values of the drag coefficient, $C_{D}$, from both CFD and the experiment are provided in Figure 4. The experimental value is shown as an orange bar with a dashed line, while the associated uncertainty is represented by the orange shaded region. The reference LES value is within 1% of the experimentally measured value, which shows a good agreement between the two methods.

For the RANS methods, both the SST method and the EBRSM exhibited closer agreement with the reference result with increasing refinement, although a 3% offset remained between the finest mesh results and the experimental value. The M mesh SA model result was close to the experimental value, but as shown in the following section, this was fortuitous since it predicted a different wake flow structure (see Section 3.3.2). For the HRL methods, the $C_{D}$ difference between the two SLSs was less than 1%, with both showing a diminishing reduction as the mesh size increased beyond the C mesh, highlighting the convergence of the solution. Meanwhile, the results from the three coarse meshes (C-WM, C-nW, and C) showed that the difference between the cases with and without wake refinement was not as significant as the difference caused by the wall-modelled prism layers. The $C_{D}$ results of the SST-DDES-$\Delta_{\max}$ and SA-DDES cases are not presented since their results were in very close agreement with those of the SST-DDES-$\Delta_{\mathrm{vol}}$ case.

### 3.2. Flow Structure

#### 3.2.1. Overview

**Figure 5:**
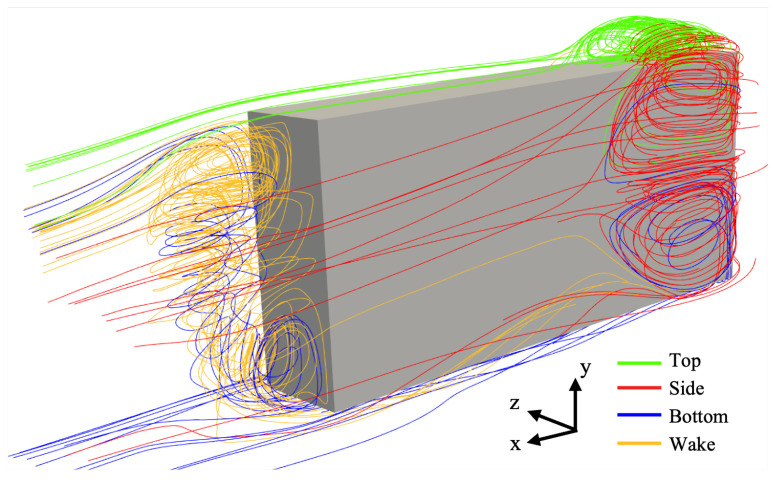
Streamline around the wagon viewed from the side rear, calculated by LES.

Based on the LES results, Figure 5 illustrates the flow structure around the wagon using streamlines seeded from different positions. In the figure, each colour represents the streamlines originating from a specific surface. Due to the sharp 90-degree corner at the leading edge, the flow separates along both the top and side surfaces, as shown by the green (top) and red (side) lines. The separation bubble, which extends along the side surfaces (red), can be divided vertically into two parts, with a deeper separation observed towards the centre height of the geometry. In the wake region (yellow), the flow from the bottom surface exerts a stronger mixing effect with the wake flow. Specifically, the up-washing high-speed flow from the underbody (blue) first mixes with the wake flow, generating a dominant z-direction vortex at the bottom of the rear surface, extending in part to the top of the wake section. As the flow continues upward, the inertia of the up-wash decreases, and the flow becomes more influenced by the free shear layers from the top and side surfaces, forming a complex 3D structure at the top of the rear surface. In the following subsections, the analyses will focus on the separation bubble prediction over the top surface and in the wake region due to the availability of experimental data.

#### 3.2.2. Separation Prediction from Top View

**Figure 6:**
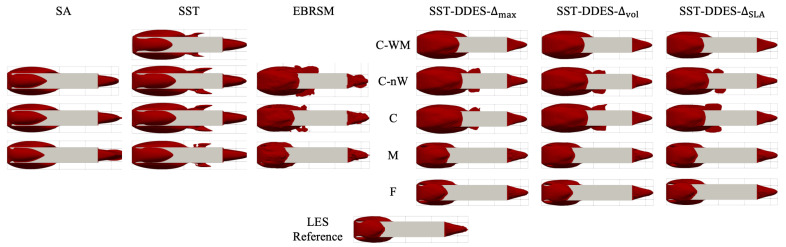
Iso-surface of the time-averaged velocity in the x-direction $|\bar{U_{x}}|=0$ from the top view, calculated by various RANS and DDES models.

Based on the understanding of the overall flow structure from the LES reference, a qualitative comparison between turbulence models and mesh resolutions was performed, focusing on the separation regions around the geometry, as shown in Figure 6, which displays the iso-surface of the time-averaged velocity in the x-direction, $|U_{x}|=0$.

According to the figures, both the SA and SST models demonstrated mesh convergence. However, neither model successfully predicted the flow structure. Both models overpredicted the extent of the separation region compared to the LES reference. Moreover, their separation bubbles on the top and side surfaces were somewhat independent of each other, i.e., they interacted less with each other when compared to the scale-resolving results. In contrast, the EBRSM was more sensitive to the mesh resolution, likely due to an unintended feature of the model, which, under certain conditions, was observed to effectively reduce levels of modelled turbulence with refinement. Meanwhile, it is also notable that some RANS models incorrectly predicted a separation region on the floor, observed as spurious features at around midway along the geometry in Figure 6.

For the DDES method, the results demonstrated significant similarities to the LES reference across all mesh resolutions, despite variations in the underlying approaches and SLSs. In terms of mesh sensitivity, the DDES results showed substantial improvement when the mesh resolution was increased from the C mesh to the M mesh, resulting in a smaller separated region. With respect to the LES reference result, the C-WM mesh provided an improved prediction compared to the other two coarse meshes (C and C-nW) in that the spurious structures around x/L=1/2 were eliminated. This suggests that the use of the wall function may actually be beneficial in this instance.

### 3.3. Velocity Field

#### 3.3.1. Comparison Between RANS and HRL Methods

**Figure 7:**
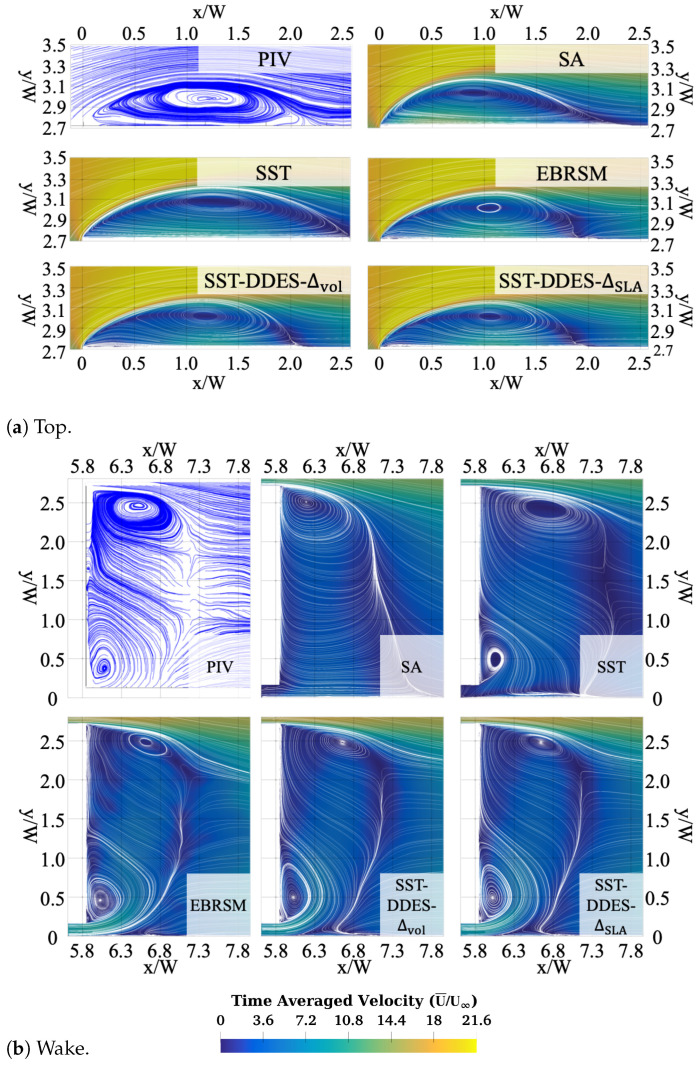
Time-averaged velocity field calculated by different models on the symmetric (z=0) plane: (**a**) over the top surface and (**b**) in the wake region.

The time-averaged flow structures on the x–y symmetric plane (z=0) from both PIV and CFD results are presented in Figure 7. All CFD results were calculated with the M mesh. Three RANS models and the SST-DDES models with $\Delta_{\mathrm{vol}}$ and $\Delta_{\mathrm{SLA}}$ were used for the comparison. According to the PIV results, for the top region (Figure 7a), there was a recirculation zone caused by separation at the LE. Both EVMs failed to correctly predict the recirculation region shape. The SA incorrectly modelled the lower part of this region where the wake interacts with the stationary boundary, while the SST overpredicted its size by 25% compared to the PIV results. Such observations have commonly been observed in the literature for similar flows and are likely due to the lack of a turbulence-resolving capability for EVMs (see, e.g., [7,36]).

On the other hand, the EBRSM and the DDES method showed a better alignment with the PIV results. However, relaminarisation effects made the EBRSM more difficult to average, as evidenced by the bumpy streamlines, which makes it tricky to apply in industrial applications. A modest but consistent improvement was visible when using $\Delta_{\mathrm{SLA}}$ instead of $\Delta_{\mathrm{vol}}$. In particular, these improvements manifested in a smaller recirculation zone and a more upstream reattachment point. In general, the DDES results demonstrated a notable and consistent advantage in flow field prediction compared to the RANS results.

#### 3.3.2. Comparison Between SLS Variants

**Figure 8:**
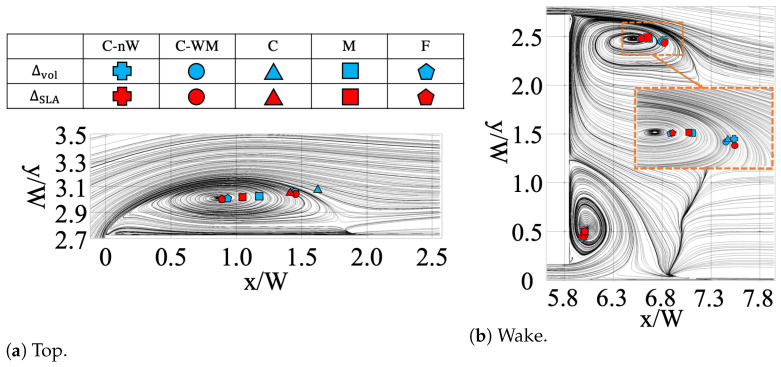
Vortex centres of SST-DDES with different SLSs on the symmetric (z=0) plane: (**a**) over the top surface and (**b**) in the wake region.

As shown in Figure 8, the vortex centres over the top surface and in the wake region were calculated based on the velocity field on the symmetric (z=0) plane and then compared with each other. The LES reference result was used to generate the streamlines shown in the background. The mesh convergence is clearly shown in both regions, similar to what was discussed in previous sections. In the top region (Figure 8a), the values of $\Delta_{\mathrm{SLA}}$ were generally observed to be closer to the LES reference across the tested meshes, underscoring the capability of the SLA approach. As mesh refinement progressed from C (triangle) to F (pentagon), the difference between the two SLSs decreased, suggesting a beneficial effect of mesh refinement. Notably, the SLA on the finest mesh (F-$\Delta_{\mathrm{SLA}}$) aligned more closely with the LES reference than F-$\Delta_{\mathrm{vol}}$, demonstrating SLA’s effectiveness in leveraging flow-informed strategies. This performance, especially for moderate and fine mesh resolutions, shows SLA’s potential suitability for industrial applications, where such refinement levels are frequently employed.

In the wake region (Figure 8b), the vortex locations were less sensitive to the mesh and turbulence models than in the top region. It is clear that the variation on the bottom side was less influenced by meshes and SLSs than the top side, as the former is mainly controlled by the high-speed up-wash flow from underneath the geometry. In contrast, the locations of the top vortices were influenced by the free shear layer shedding from the top surface, whose accuracy is more dependent on the choice of turbulence models and grid sizes. In the zoomed-in view, the difference between the meshes is minimal when the mesh size reaches M, which aligns with the trend of the mesh convergence shown in the $C_{D}$ and $C_{p}$ plots.

### 3.4. Pressure Coefficient

**Figure 9:**
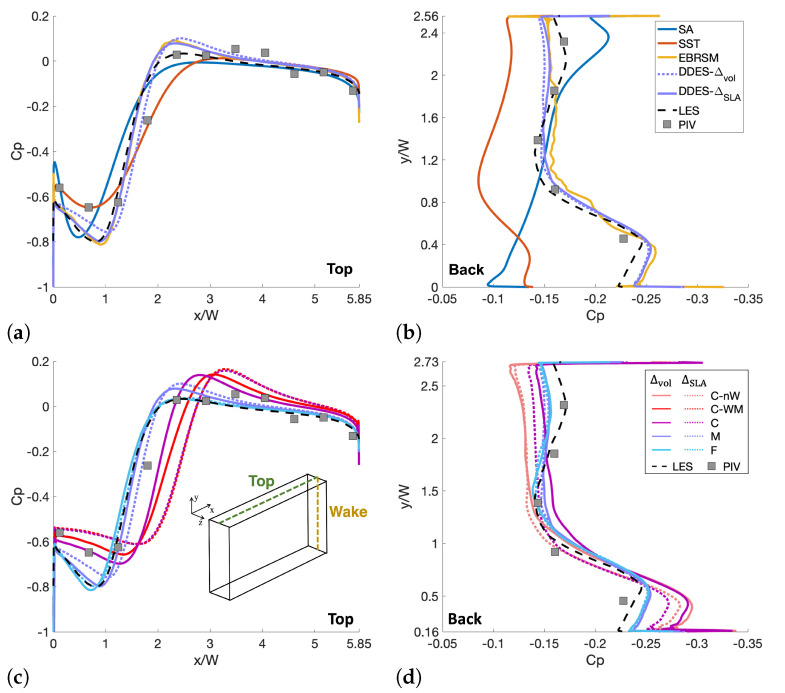
Pressure coefficients along the centrelines of the (**a**) top surface and (**b**) back surface for the RANS and DDES turbulence models with the M mesh, and the (**c**) top surface and (**d**) back surface for the SST-DDES model with different meshes and SLS models.

Figure 9a compares the results calculated using different turbulence models with the M mesh over the centreline (z=0) of the top surface. SST-DDES with $\Delta_{\mathrm{vol}}$ and $\Delta_{\mathrm{SLA}}$ are used to represent the HRL methods. Figure 9c provides the same for the DDES variants on different meshes. Figure 9b,d provide the same for the rear wake.

One can observe that there is less spread in the former compared to the latter, although the RANS EVMs predict a different form of pressure recovery. When cross-referenced with Figure 7a, it indicates that, despite a reasonable prediction of $C_{p}$, the flow structure predicted by the SST model is larger than it should be, as discussed in Section 3.3.1. Meanwhile, an inspection shows that the EBRSM returns unphysically low values of turbulence, reinforcing observations that the model has effectively reverted to a scale-resolving method. Thus, the results are similar to those from the DDES methods. The nature of the separation on the top surface is exactly the type of flow that $\Delta_{\mathrm{SLA}}$ is designed to address, reducing modelled turbulence and, therefore, turbulent viscosity in a way that maximises scale resolution.

Figure 9b shows somewhat erroneous behaviour reported by both the SA and SST models. The form of $C_{p}$ predicted by the SST model has a similar pattern but is underpredicted compared to the experimental and scale-resolving results, leading to a smaller $C_{D}$. In contrast, the SA model underpredicts the separation in the top region and overpredicts it in the bottom region, indicated as an overprediction and underprediction of $C_{p}$, respectively. Fortuitously, the overprediction cancels out the underprediction, resulting in a value of $C_{D}$ that compares favourably with both the experimental and LES results.

In Figure 9c,d, the comparison switches to the predictions from the SST-DDES model for different meshes and SLS models. The results for the C-nW mesh are not presented in this figure since its mesh is identical to the C mesh over the top surface. According to the plot, the CFD results show a clear trend of mesh convergence towards the finest mesh (light green). Meanwhile, the agreement between the experimental and CFD results with fine meshes is generally good, although almost all CFD cases overpredict the low peak at x/W≈1. The results from the M mesh and above are very close, while the results from the C-WM and C meshes behave as outliers. For these two meshes, the $\Delta_{\mathrm{SLA}}$ results are closer to the finer mesh and experimental results compared with the other two SLS variants.

In Figure 9d the C-nW mesh is plotted for comparison, but the C-WM mesh is excluded since its wake refinement level is identical to that of the C mesh. As for the previous plot, the mesh convergence towards the LES results is clear, and the CFD results show good agreement with the experimental results. The impact of the SLS variant is notably lower in this region since the turbulence is already fully three-dimensional and operating entirely in the LES mode (see Section 3.6).

### 3.5. Resolved Reynolds Stress

**Figure 10:**
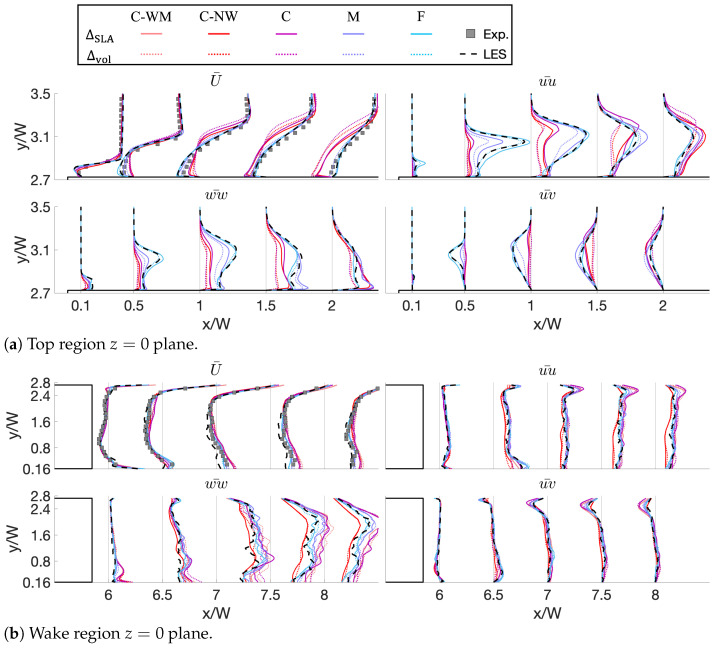
Comparison of time-averaged streamwise velocity and resolved Reynolds stress profiles over the plane z=0 (**a**) at the forward top corner and (**b**) in the wake region. Results for SST-DDES with different SLS and different meshes. The *x* coordinate of $\bar{U_{x}}$ is calculated by $x/W=\bar{U_{x}}/U_{\infty }\times F_{U_{x}}$, and the *x* coordinate of $\bar{u_{i}u_{j}}$ is calculated by $x/W=\bar{u_{i}u_{j}}/U_{\infty }^{2}\times F_{u_{i}u_{j}}$, with $F_{U_{x}}=0.25,F_{u_{i}u_{j}}=2.5$ in (**a**) and $F_{U_{x}}=0.6,F_{u_{i}u_{j}}=6$ in (**b**).

A more quantitative comparison of different meshes and SLSs is shown in Figure 10, where the time-averaged streamwise velocity ($\bar{U_{x}}$) and velocity fluctuations ($\bar{u_{i}u_{j}}$) are plotted along the symmetric plane (z=0). As above, the focus is placed on both the top and wake regions. The time-averaged velocity profiles are plotted in the top-left corner of each figure, and the mesh’s convergence towards the LES reference (black dash) and experimental (grey square) data is clearly shown.

In Figure 10a, all $\bar{u_{i}u_{j}}$ plots show that, for the top separation region, $\Delta_{\mathrm{SLA}}$ (solid) has a higher level of resolved Reynolds stress at the initial stage of the shear layers, which meets the expectation of this SLS. Also, it is notable that, as shown in Figure 10b, in the entire wake region, the Reynolds stress level does not display significant sensitivity to different meshes and turbulence models, except in a minor way for regions close to the shear layer. Overall, the flow structure in the wake is less sensitive to the choice of turbulence model and mesh refinement level.

On the contrary, the mesh has an obvious influence when a high-fidelity turbulence model is applied, especially in the top region. At the initial stage of the separation bubble, the $\bar{uu}$ and $\bar{ww}$ plots show that the Reynolds stress level is very sensitive to the mesh resolution. Such a difference results in the maximum Reynolds stress values of coarse meshes being located more downstream than for finer ones. However, it should be noted that, although the locations of the maximum values are different, their intensities are at a similar level, given a maximum 400% grid size difference.

### 3.6. Turbulence Kinetic Energy

**Figure 11:**
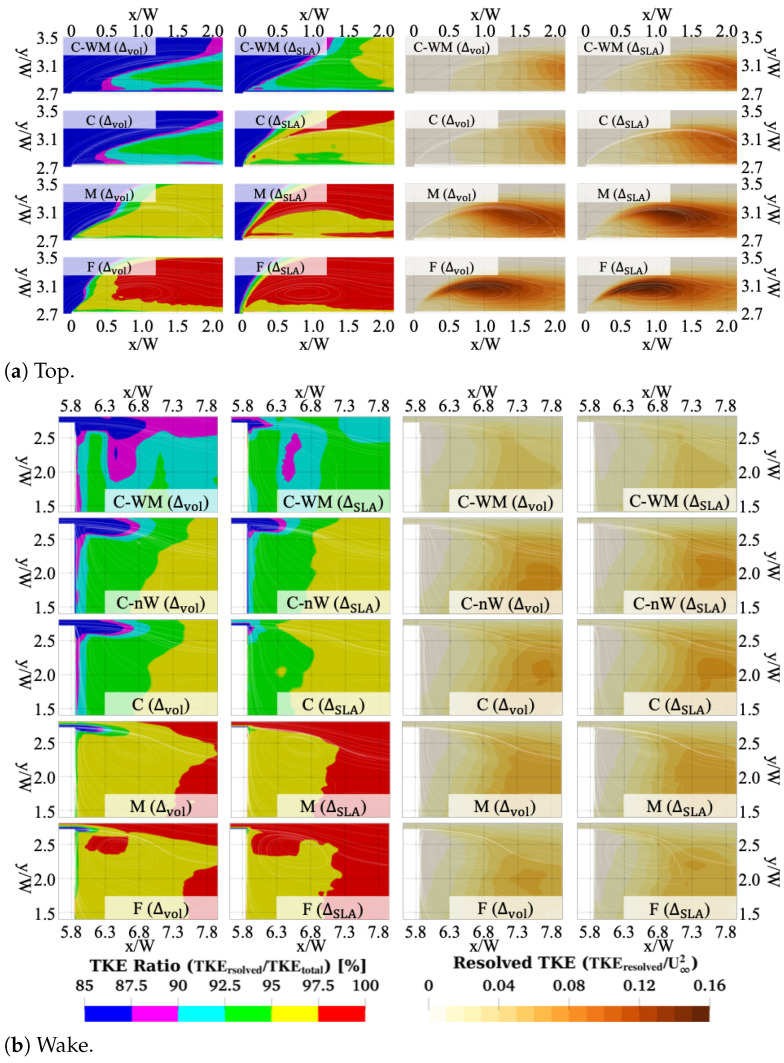
(Left two columns) TKE ratio, defined as the ratio between the resolved TKE and the total TKE (${\mathrm{TKE}}_{\mathrm{res}}/{\mathrm{TKE}}_{\mathrm{tot}}$) for $\Delta_{\mathrm{vol}}$ and $\Delta_{\mathrm{SLA}}$; (right two columns) non-dimensionalised resolved TKE, normalized by the free-stream velocity (${\mathrm{TKE}}_{\mathrm{res}}/U_{\infty }^{2}$). Results are shown (**a**) over the top surface and (**b**) in the wake region, calculated using SST-DDES with different mesh resolutions.

A comparison of the TKE across the different meshes is shown in Figure 11, where the left two columns display the ratio of the resolved TKE to the total TKE (TKE ratio, ${\mathrm{TKE}}_{\mathrm{res}}/{\mathrm{TKE}}_{\mathrm{tot}}$) for $\Delta_{\mathrm{vol}}$ and $\Delta_{\mathrm{SLA}}$, respectively. The right two columns show the resolved TKE level, normalized by the free-stream velocity (${\mathrm{TKE}}_{\mathrm{res}}/U_{\infty }^{2}$). All results are presented on the symmetry plane (z=0). The resolved TKE is computed as ${\mathrm{TKE}}_{\mathrm{res}}=0.5\left(\bar{\mathrm{uu}}+\bar{\mathrm{vv}}+\bar{\mathrm{ww}}\right)$.

In general, the ratio of the resolved TKE to the total TKE was observed to increase with mesh refinement, as expected. Coarser meshes struggled to resolve sufficient levels of turbulence in the early stages of the shear layer, resulting in a delayed peak in TKE. However, the resolved levels of TKE were notably higher when using $\Delta_{\mathrm{SLA}}$ than $\Delta_{\mathrm{vol}}$, and the transition to fully resolved turbulence was accelerated, reducing the extent of the ‘grey-area’ region. These observations correlate well with the behaviour in the resolved Reynolds stress profiles observed in the previous section.

For the wake region, the TKE ratio exhibited similar behaviour to the top region, but the resolved TKE level was not as strongly correlated with the mesh refinement level. The C-nW results showed the lowest resolved TKE level due to its poor resolution, but the C mesh showed the highest resolved TKE level compared with the other cases.

### 3.7. Taylor Microscale and Ratio

It is common practice to evaluate the ratio of eddy to laminar viscosity, as well as the ratio of resolved to total TKE when assessing mesh quality in HRL methods. Another possibility exists that uses an approximation for the Taylor microscale (λ), defined as the scale of motion at which fluctuations become isotropic [53,54]. This is of particular importance in HRL methods since RANS methods are expected to perform reasonably well under such conditions, i.e., as opposed to predicting the interaction of complex anisotropic turbulent structures.

In DDES simulations, the Taylor microscale can be approximated using the modelled specific dissipation rate $\omega_{\mathrm{mod}}$ as follows [54]:

(5) $$\lambda_{\mathrm{mod}}=\sqrt{10\frac{\nu \times k_{\mathrm{mod}}}{\varepsilon_{\mathrm{mod}}}}=\sqrt{\frac{10\nu }{C_{\mu }\times \omega_{\mathrm{mod}}}}.$$

In Equation (5), the functional dependence is on the specific dissipation rate ω, instead of *k*.

**Figure 12:**
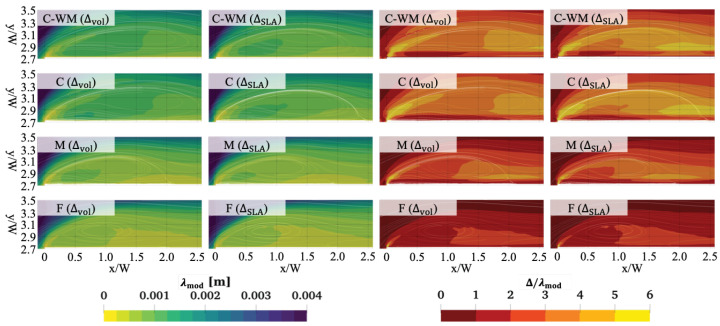
(**Left**) Taylor microscale λ and (**right**) ratio of SLS to Taylor microscale, Δ/λ, over the top surface, calculated using the SST-DDES-$\Delta_{\mathrm{vol}}$ and $\Delta_{\mathrm{SLA}}$ models with different meshes.

The Taylor microscale over the top surface is plotted on the left side of Figure 12. In the figure, we can observe that the differences between the three SLSs are less pronounced than in the eddy viscosity ratio and TKE ratio plots. More importantly, the results from lower mesh resolutions (e.g., C-$\Delta_{\mathrm{SLA}}$) are consistently less accurate than those from higher-resolution meshes. As the mesh is refined, we observe a decrease in the Taylor microscale, indicating that finer meshes capture more detailed turbulence structures.

We also non-dimensionalised the Taylor microscale, $\lambda_{\mathrm{mod}}$, by calculating the ratio of the SLS (either $\Delta_{\mathrm{vol}}$ or $\Delta_{\mathrm{SLA}}$) to $\lambda_{\mathrm{mod}}$, as shown on the right side of Figure 12, which may be helpful in developing best practice guidelines for the use of DDES in such cases. Based on the observations in Section 3.3.2, the results indicate that satisfactory resolution of the turbulence requires a mesh resolution where Δ/λ∼3 or less in the immediate vicinity of the corner.

## 4. Conclusions

In this study, both experimental and computational methods were employed to predict the flow field around a complex geometry. The experimental results aligned well with the reference data, with all utilized CFD methods differing by less than 10% from the reference.

Ten turbulence models and six meshes were tested in the computational part to assess the capability and mesh sensitivity of the RANS and HRL methods. A comparison of the lift coefficient and mean flow field showed that most of the results fell within the uncertainty range of the experimental results, although this was, in some cases, observed to be fortuitous. A more detailed comparison of the flow structure showed that the standard RANS models, including SA and SST, incorrectly predicted the size and shape of the separation bubble. While the DDES approaches were able to provide significantly improved predictions, a clear dependence on mesh resolution was observed. This underscores the importance of employing adequate mesh resolutions with these methods. The influence of the mesh refinement, model, and SLS was more pronounced for the top surface separation than for the wake region due to the nature of the turbulence present in this region.

The comparison of SLS selections demonstrated that $\Delta_{\mathrm{vol}}$ and $\Delta_{\max}$ exhibited similar behaviour when applied to octree meshes, while $\Delta_{\mathrm{SLA}}$ proved more effective at resolving turbulence, bringing the results closer to the reference when the mesh resolution was insufficient. This finding underscores the robustness and practical relevance of the SLA approach for industrial applications, where moderate mesh refinements are commonly used. However, the improvement in accuracy gained is less than what could be achieved with further refinement.

Finally, the Taylor microscale was explored as a means of approximating the mesh requirement using a precursor RANS result. Based on the results of this study, it was observed that the ratio of the SLS to the Taylor microscale (Δ/λ) should not exceed 3, particularly in regions where the DDES switches from RANS to LES modes. While there is some reason to expect that a value or range of values exists that is independent of the flow, this clearly requires more extensive testing.

## Figures and Tables

**Table 1:** Test matrix of this study.

	Turbulence Model								
	RANS			SA-DDES			SST-DDES		
Mesh	SA	SST	EBRSM	Δ_vol_	Δ_max_	Δ_SLA_	Δ_vol_	Δ_max_	Δ_SLA_
C-WM		√		√	√	√	√	√	√
C-noWake	√	√	√	√	√	√	√	√	√
C	√	√	√	√	√	√	√	√	√
M	√	√	√	√	√	√	√	√	√
F				√	√	√	√	√	√
LES	Only used for LES benchmark generation.								

**Table 2:** Mesh setup.

Mesh	Cell Count (Million)	Wagon Surface Mesh Size (mm)	$y_{\mathrm{wagon}}^{+}$	$x_{\mathrm{wagon}}^{+}$	$z_{\mathrm{wagon}}^{+}$	$y_{\mathrm{floor}}^{+}$
C-WM	3.2	2	26.6	53.2	53.2	32.7
C-noWake	3.4	2	0.44	44.1	44.1	0.58
C	5.4	2	0.45	44.8	44.8	0.48
M	18.7	1	0.43	21.5	21.5	0.45
F	36.0	1	0.44	22.8	22.8	0.45
LES ^1^	89.4	0.5	0.52	12.9	12.9	0.51

^1^ Based on LES simulation with *k* equation model and $\Delta_{\mathrm{vol}}$.

## Data Availability

The data presented in this study are available on request from the corresponding author.

## Appendix A. Shear-Layer-Adapted Subgrid Length Scale

The idea of the shear-layer-adapted subgrid length scale, $\Delta_{\mathrm{SLA}}$, is based on the SLS ${\tilde{\Delta }}_{\omega }$, previously developed by Mockett et al. [22]: For a cell located at r with vertices at $r_{n}$ (n=1,2,...,8 for a hexahedral mesh), the operator ${\tilde{\Delta }}_{\omega }$ is defined as

(A1) $${\tilde{\Delta }}_{\omega }=\frac{1}{\sqrt{3}}\max_{n,m=1,2,...,8}∥\left(I_{n}-I_{m}\right)∥,$$

where $I_{n}=n_{\omega }\times \left(r_{n}-r\right)$ and $n_{\omega }$ is the unit vector of the vorticity vector. The operator $\Delta_{\mathrm{SLA}}$ is then defined as

(A2) $$\Delta_{\mathrm{SLA}}={\tilde{\Delta }}_{\omega }\;F_{KH}\left(<VTM>\right),$$

where the function $F_{KH}\left(<VTM>\right)$ is used to suppress the $\Delta_{\mathrm{SLA}}$ in the quasi-2D region in order to permit a more accurate representation of the Kelvin–Helmholtz (KH) instability [18]. Specifically, the VTM, Vortex Tilting Measure, is defined as

(A3) $$VTM=\frac{\sqrt{6}∥\left(\hat{S}\cdot \omega \right)\times \omega ∥}{\omega^{2}\sqrt{3\mathrm{tr}\left(\hat{S^{2}}\right)-{\mathrm{tr}}^{2}\left(\hat{S}\right)}},$$

where $\hat{S}$ is the strain rate tensor, with $\hat{S}=1/2\left(\nabla u+\nabla u^{T}\right)$, and tr means the trace of the tensor. The angle bracket in Equation (A2) denotes that the VTM should be averaged over the current and closest cells due to its severe spatial oscillation caused by velocity gradient calculation. Meanwhile, $F_{KH}$ is defined as

(A4) $$F_{KH}\left(x\right)=\max\left\{F_{KH}^{\min},\;\min\left[F_{KH}^{\max},\;F_{KH}^{\min}+\frac{F_{KH}^{\max}-F_{KH}^{\min}}{a_{2}-a_{1}}\left(x-a_{1}\right)\right]\right\},$$

where $F_{KH}^{\max},\;F_{KH}^{\min},\;a_{1},\;a_{2}$ are empirical values that are set to 1,0.1,0.15,0.3, respectively.
